# Comparison of incident hypertension between SGLT2 inhibitors vs. DPP4 inhibitors

**DOI:** 10.1038/s41440-024-01649-z

**Published:** 2024-04-10

**Authors:** Yuta Suzuki, Hidehiro Kaneko, Akira Okada, Jin Komuro, Katsuhito Fujiu, Norifumi Takeda, Hiroyuki Morita, Junya Ako, Akira Nishiyama, Yuichiro Yano, Masaki Ieda, Koichi Node, Hideo Yasunaga, Issei Komuro

**Affiliations:** 1 The Department of Cardiovascular Medicine, The University of Tokyo, Tokyo, Japan; 2 Center for Outcomes Research and Economic Evaluation for Health, National Institute of Public Health, Saitama, Japan; 3 The Department of Advanced Cardiology, The University of Tokyo, Tokyo, Japan; 4 Department of Prevention of Diabetes and Lifestyle-Related Diseases, Graduate School of Medicine, The University of Tokyo, Tokyo, Japan; 5 Department of Cardiology, Keio University School of Medicine, Tokyo, Japan; 6 Department of Cardiovascular Medicine, Kitasato University School of Medicine, Sagamihara, Japan; 7 Department of Pharmacology, Faculty of Medicine, Kagawa University, Kagawa, Japan; 8 Department of General Medicine, Juntendo University Faculty of Medicine, Tokyo, Japan; 9 Department of Family Medicine and Community Health Duke University Durham NC, Durham, NC USA; 10 Department of Cardiovascular Medicine, Saga University, Saga, Japan; 11 Department of Clinical Epidemiology and Health Economics, School of Public Health, The University of Tokyo, Tokyo, Japan; 12 International University of Health and Welfare, Tokyo, Japan

**Keywords:** SGLT2 inhibitors, Hypertension, Diabetes, Epidemiology

## Abstract

Although several randomized clinical trials have reported the potential benefit of sodium-glucose cotransporter 2 inhibitors (SGLT2i) in reducing blood pressure (BP), whether SGLT2i can reduce incident hypertension is unknown. We analyzed individuals with diabetes who were newly prescribed SGLT2i or dipeptidyl peptidase 4 inhibitors (DPP4i) in a large-scale epidemiological database. The primary outcome was the incidence of hypertension. A propensity score matching algorithm was employed to compare the subsequent development of hypertension between the SGLT2i and DPP4i groups. After propensity score matching, 5708 well-balanced pairs of SGLT2i and DPP4i users were identified. SGLT2i administration was associated with a reduced risk of hypertension (HR 0.91, 95% CI: 0.84–0.97). The advantage of SGLT2i use over DPP4i use for incident hypertension was generally consistent in several sensitivity analyses, and subgroup analyses showed that SGLT2i use was significantly associated with a lower risk of hypertension in men, patients with baseline HbA1c of <7.5%, and baseline systolic blood pressure ≥127 mmHg. Our investigation using nationwide real-world data demonstrated the potential advantage of SGLT2i over DPP4i in reducing the development of hypertension in individuals with diabetes.

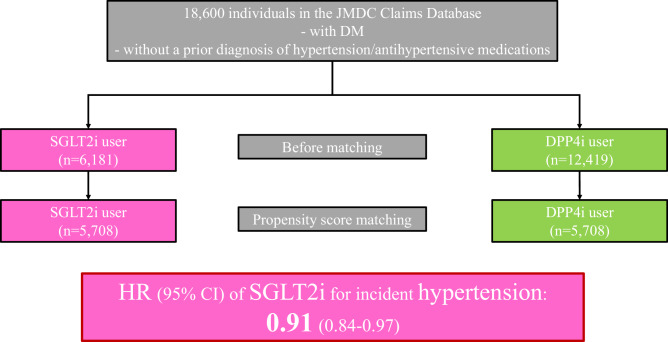

## Introduction

Sodium-glucose cotransporter 2 inhibitor (SGLT2i) was initially developed as a novel medication for diabetes, functioning by impeding glucose reabsorption in the proximal tubule of the kidney, thereby enhancing the excretion of glucose through the urine and leading to improved glycemic control [1–3]. Recent clinical trials have revealed the substantial cardiovascular and kidney protective benefits of SGLT2i [4–9]. Several randomized controlled trials (RCTs) have also indicated the potential of SGLT2i to have blood pressure (BP) lowering effects [10–14]. However, the reported reduction in BP is around 3–4 mmHg for systolic BP (SBP), and it remains unclear whether SGLT2i could effectively reduce incident hypertension. Hypertension not only independently increases the risk of cardiovascular events but also occurs frequently as a complication of diabetes [15]. Further, it is well known that hypertension and diabetes additively elevate the risk of cardiovascular events. Therefore, if SGLT2i can decrease the risk of developing hypertension, it should be recognized as an additional benefit and potential indication. In this study, we analyzed a nationwide epidemiological database and examined whether SGLT2i use is associated with a lower risk of developing hypertension.

Point of view

- Clinical relevance
  In individuals with diabetes, the introduction of SGLT2 inhibitors could reduce the risk of developing hypertension compared to DPP4 inhibitors.
- Future direction
  Well-designed prospective trials are needed to test whether SGLT2 inhibitors are effective in preventing the development of hypertension.
- Consideration for the Asian population
  The coexistence of diabetes and hypertension represents a significant clinical challenge in Asian countries, and this study holds valuable implications for the clinical application of SGLT2 inhibitors within Asian populations.

## Materials and methods

Anonymized data are publicly available for purchase from JMDC, Inc.

### Study population

**Fig. 1:**
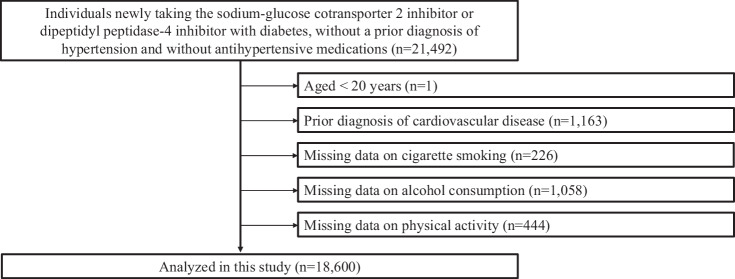
Flowchart. We extracted the data of 21,492 individuals with diabetes, without a prior diagnosis of hypertension and without antihypertensive medications, who newly initiated sodium-glucose cotransporter 2 inhibitors (SGLT2i) or dipeptidyl peptidase-4 inhibitors (DPP4i). Among 21,492 individuals, we excluded participants for the following reasons: aged <20 years (*n* = 1); a prior diagnosis of cardiovascular diseases such as myocardial infarction, angina pectoris, stroke, heart failure, and atrial fibrillation (*n* = 1163); missing cigarette smoking data (*n* = 226), missing alcohol consumption data (*n* = 1058), and missing physical activity data (*n* = 444) Finally, 18,600 individuals were included in this study

This retrospective cohort study used the JMDC Claims Database, a large-scale administrative claims database [16–18] that uses data from annual employee health checkups which are a legal requirement in Japan. The JMDC includes annual health checkup data (e.g., blood tests and anthropometric measurements) and health insurance records between 2005 and 2022. Medical diagnoses were coded according to the International Classification of Diseases, 10th revision (ICD-10). This study adopted an active comparator, a new user design to account for confounding by indication and unmeasured confounders (Supplementary Fig. 1) [19]. The active comparator group was composed of individuals who initiated treatment with dipeptidyl peptidase-4 inhibitors (DPP4i). Considering the relatively high prescription rate of DPP4i for individuals with diabetes in Japan [20], individuals who were newly prescribed DPP4i were set as the control group in this study. We extracted the data of 21,492 individuals with diabetes (ICD-10 codes E10–E14), without a prior diagnosis of hypertension (ICD-10 code: I10-I15) and without antihypertensive medications, who had newly initiated SGLT2i or DPP4i treatment. To exclude previous users, new usage was defined as starting either drug class in those who had never used either drug class in the past year. Among the 21,492 individuals, we excluded participants for the following reasons: age <20 years (*n* = 1); prior diagnosis of cardiovascular diseases such as myocardial infarction, angina pectoris, stroke, heart failure, and atrial fibrillation (*n* = 1163); missing cigarette smoking data (*n* = 226); missing alcohol consumption data (*n* = 1058); and missing physical activity data (*n* = 444). Ultimately, 18,600 individuals were included in this study (Fig. 1).

### Ethics

This study was approved by the Ethics Committee of the University of Tokyo (approval number:2018-10862), and informed consent was waived because all data included in the JMDC Claims Database were anonymized and de-identified.

### Measurements and definitions

We obtained the following data from the health checkups: body mass index (BMI), BP, laboratory data (fasting plasma glucose, hemoglobin A1c [HbA1c], low-density lipoprotein cholesterol, high-density lipoprotein cholesterol, triglycerides), cigarette smoking (current or noncurrent/never), alcohol consumption (daily or not every day), and physical activity (active or inactive). Cigarette smoking and alcohol consumption were assessed using a self-report questionnaire during health checkups. Physical inactivity was defined as not exercising for 30 min ≥twice a week or not walking for more than an hour per day. Based on the ICD-10 codes, we obtained data on the presence of diabetic nephropathy (ICD-10 codes E102, E112, E122, E132, and E142), diabetic retinopathy (ICD-10 codes E103, E113, E123, E133, and E143), and diabetic neuropathy (ICD-10 codes E104, E114, E124, E134, and E144) at the date of prescription of SGLT2i or DPP4i. Data on concomitant medications at the prescription date of SGLT2i or DPP4i were extracted from the administrative claims records.

### Propensity score matching

A propensity score-matching algorithm was used to generate a matched cohort to compare the benefits of SGLT2i and DPP4i use. We estimated the propensity scores of the SGLT2i users using a logistic regression model. To estimate the propensity score, we included the following variables: age, sex, BMI, SBP, diastolic blood pressure (DBP), fasting plasma glucose, HbA1c, low-density lipoprotein cholesterol, high-density lipoprotein cholesterol, triglycerides, cigarette smoking, alcohol consumption, physical inactivity, diabetic nephropathy, diabetic retinopathy, diabetic neuropathy, use of medications (insulin, glucagon-like peptide-1 receptor agonist, biguanide, sulfonylurea, α-glucosidase inhibitor, thiazolidine, glinide, and statins), and year of SGLT2i or DPP4i prescription. SGLT2i and DPP4i users were matched using a 1:1 matching protocol (caliper width equal to 0.2 standard deviations of the logit score).

### Outcomes

The primary outcome was incident hypertension (ICD-10 codes I10-I15). We followed the study participants from the index date (i.e., initiation of SGLT2i or DPP4i) to the incidence of hypertension, discontinuation of insurance, death, or study end date (May 2022). We did not complete the follow-up even if an SGLT-2i or DPP4i is initiated and the other is prescribed additionally.

### Statistical analysis

Descriptive statistics were reported as median (interquartile range) and number (percentage). Standardized mean differences were used to compare the clinical characteristics of SGLT2i and DPP4i users. The hazard ratios (HRs) and 95% confidence intervals (95% CI) of the incidence of hypertension in SGLT2i users versus DPP4i users were estimated using a Cox proportional hazards regression model. We also conducted subgroup analysis by age (≥50 and <50 years), sex, median BMI value (≥26.4 and <26.4 kg/m^2^), median HbA1c level (≥7.5 and <7.5%), and median SBP value (≥127 and <127 mmHg).

Seven sensitivity analyses were conducted. First, we examined the incidence of hypertension in individuals who continued to use SGLT2i or DPP4i for >3 months. Second, we analyzed individuals with a diagnosis of type 2 diabetes (ICD-10 code E11). Third, we excluded individuals with SBP ≥ 140 mmHg or DBP ≥ 90 mmHg at the initial health checkup. Fourth, the outcome was redefined as a diagnosis of hypertension with a prescription for antihypertensive medications (World Health Organization Anatomical Therapeutic Chemical [WHO-ATC] codes C02, C03, C04, C07, C08, and C09) in the months before and after the diagnosis of hypertension. Fifth, we excluded individuals with glucagon-like peptide-1 receptor agonist at the index date. Sixth, we repeated the primary analyses after excluding individuals who had any antidiabetic medications at the index date. Finally, we performed an analysis using overlap weighting to balance the exposure groups (SGLT2i and DPP4i). Overlap weights were defined as 1−propensity score among SGLT2i users and as the propensity score among DPP4i users. All statistical analyses were performed using STATA version 17 (StataCorp LLC, College Station, TX, USA).

## Results

### Clinical characteristics

Table 1 presents the baseline clinical characteristics of the study participants before and after propensity score matching. After 1:1 propensity score matching, 5708 pairs were created. The individual distributions were well balanced between SGLT2i and DPP4i users. The median age was 50 (44–55) years for SGLT2i users and 49 (43–55) years for DPP4i users. In addition, 4471 (78.3%) individuals were men in SGLT2i users, and 4487 (78.6%) individuals were men in DPP4i users. The median SBP and DPB were 127 (118–138) and 81 (74–88) mmHg, respectively, in SGLT2i users, and 128 (119–138) and 81 (74–88) mmHg, respectively, in DPP4i users.

**Table 1:** Baseline characteristics

	Before propensity score matching			After propensity score matching		
	DPP4i (*n* = 12,419)	SGLT2i (*n* = 6181)	SMD	DPP4i (*n* = 5708)	SGLT2i (*n* = 5708)	SMD
Age, years	52 (46–58)	49 (43–55)	−0.329	49 (43–55)	50 (44–55)	0.018
Men, n (%)	9873 (79.5)	4806 (77.8)	−0.043	4487 (78.6)	4471 (78.3)	−0.007
BMI, kg/m^2^	25.7 (23.1–28.8)	28.0 (25.2–31.4)	0.501	27.8 (24.9–31.3)	27.8 (25.1–30.9)	−0.029
SBP, mmHg	127 (118–138)	128 (118–138)	0.012	128 (119–138)	127 (118–138)	−0.015
DBP, mmHg	80 (73–88)	81 (74–88)	0.048	81 (74–88)	81 (74–88)	−0.01
Cigarette smoking, *n* (%)	4667 (37.6)	2166 (35.0)	−0.053	2009 (35.2)	2031 (35.6)	0.008
Alcohol consumption, *n* (%)	2690 (21.7)	1025 (16.6)	−0.129	977 (17.1)	998 (17.5)	0.01
Physical inactivity, *n* (%)	6970 (56.1)	3581 (57.9)	0.037	3276 (57.4)	3296 (57.7)	0.007
Comorbidity						
Diabetic nephropathy, *n* (%)	620 (5.0)	501 (8.1)	0.126	392 (6.9)	410 (7.2)	0.012
Diabetic retinopathy, *n* (%)	1144 (9.2)	828 (13.4)	0.132	648 (11.4)	685 (12.0)	0.02
Diabetic neuropathy, *n* (%)	139 (1.1)	119 (1.9)	0.066	90 (1.6)	90 (1.6)	0.000
Medication						
Insulins, *n* (%)	471 (3.8)	599 (9.7)	0.237	394 (6.9)	417 (7.3)	0.016
GLP-1 receptor agonist, *n* (%)	41 (0.3)	125 (2.0)	0.157	41 (0.7)	50 (0.9)	0.018
Biguanide, *n* (%)	1900 (15.3)	1327 (21.5)	0.16	1157 (20.3)	1146 (20.1)	−0.005
Sulfonylurea, *n* (%)	390 (3.1)	174 (2.8)	−0.019	156 (2.7)	154 (2.7)	−0.002
α-GI, *n* (%)	349 (2.8)	171 (2.8)	−0.003	144 (2.5)	154 (2.7)	0.011
Thiazolidine, *n* (%)	112 (0.9)	114 (1.8)	0.081	86 (1.5)	83 (1.5)	−0.004
Glinides, *n* (%)	98 (0.8)	60 (1.0)	0.019	47 (0.8)	51 (0.9)	0.008
Statin, *n* (%)	1865 (15.0)	1119 (18.1)	0.083	1042 (18.3)	1019 (17.9)	−0.01
Laboratory Data						
Glucose, mg/dL	147 (125–190)	141 (119–181)	−0.119	141 (122–178)	141 (119–182)	0.004
HbA1c, %	7.5 (6.8–9.1)	7.4 (6.6–8.7)	−0.131	7.4 (6.7–8.6)	7.4 (6.6–8.8)	0.001
LDL-C, mg/dL	136 (113–160)	137 (114–159)	−0.006	136 (114–161)	137 (114–159)	−0.017
HDL-C, mg/dL	51 (44–61)	49 (42–58)	−0.14	49 (42–58)	50 (43–58)	0.018
Triglycerides, mg/dL	138 (93–209)	145 (99–217)	0.062	147 (101–218)	145 (99–218)	−0.003

Data are reported as medians (interquartile range) or numbers (percentage), where appropriate

*DPP-4i* dipeptidyl peptidase-4 inhibitors, *SGLT2i* sodium-glucose cotransporter-2 inhibitors, *BMI* body mass index, *SBP* systolic blood pressure, *DBP* diastolic blood pressure, *GLP-1* glucagon-like peptide 1, *α-GI* α-glucosidase inhibitor, *LDL-C* low-density lipoprotein cholesterol, *HDL-C* high-density lipoprotein cholesterol

### Risk of developing hypertension between SGLT2 and DPP4 inhibitors

**Fig. 2:**
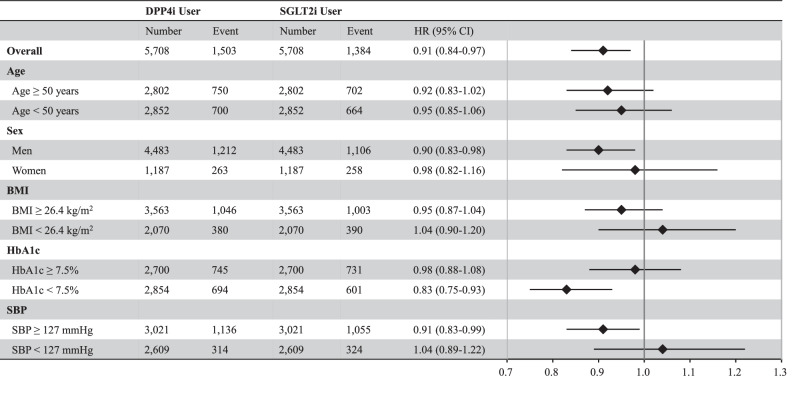
Hazard Ratio of Developing Hypertension. We performed Cox proportional hazards regression model to estimate the hazard ratio (HR) and 95% confidence interval (95% CI) of hypertension incidence with sodium-glucose cotransporter 2 inhibitors (SGLT2i) versus dipeptidyl peptidase-4 inhibitors (DPP4i) after propensity score matching. Subgroup analysis was performed by age (≥50 and <50 years), sex, median body mass index (BMI) value (≥26.4 and <26.4 kg/m^2^), median hemoglobin A1c (HbA1c) level (≥7.5 and <7.5%), and median systolic blood pressure (SBP) value (≥127 and <127 mmHg)

The mean follow-up duration was 564 ± 493 days (574 ± 499 days for SGLT2i users and 553 ± 487 days for DPP4i users). After propensity score matching, the Cox regression analysis presented that the risk of developing hypertension was reduced in individuals prescribed SGLT2i compared to those prescribed DPP4i (HR 0.91, 95% CI 0.84–0.97). Subgroup analyses stratified by age, sex, BMI, HbA1c, and SBP showed that SGLT2i use was significantly associated with a decreased risk of developing hypertension in men, a baseline HbA1c level <7.5%, and a baseline SBP ≥ 127 mmHg (Fig. 2).

### Sensitivity analyses

First, we studied 4318 pair who continued to use SGLT2i or DPP4i for >3 months. In this population, the risk of developing hypertension was lower in SGLT2i users than in DPP4i users (Supplementary Table 1). Second, we studied 3992 pairs with a prior diagnosis of type 2 diabetes. The HR (95% CI) of SGLT2i for developing hypertension was 0.93 (0.86–1.01; Supplementary Table 2). Third, we analyzed 4067 pairs after excluding individuals who had SBP ≥ 140 mmHg or DBP ≥ 90 mmHg at the initial health checkup. Even in this case scenario, the risk of developing hypertension was lower in SGLT2i users than in DPP4i users (Supplementary Table 3). Fourth, we redefined the outcome as a diagnosis of hypertension with prescription of antihypertensive medications in the month before and after diagnosis. SGLT2i users had a lower risk of developing hypertension than DPP4i users (Supplementary Table 4). Fifth, the risk of developing hypertension was lower in SGLT2i users than in DPP4i users even after excluding individuals with glucagon-like peptide-1 receptor agonist (Supplementary Table 5). Sixth, the HR and 95% CI of developing hypertension in SGLT2i users was 0.93 (0.86–1.01) compared to that in DPP4 inhibitor users after excluding individuals who had any antidiabetic medications at the index date (Supplementary Table 6). Finally, the risk of developing hypertension was lower in the SGLT2i users than in the DPP4i users after the overlap weighting procedure (Supplementary Table 7).

## Discussion

Our analysis used a nationwide large-scale health check-up and insurance claims dataset, including approximately 20,000 individuals with diabetes, and compared the subsequent risk of developing hypertension between SGLT2i and DPP4i users after propensity score matching. SGLT2i administration was associated with a decreased risk of developing hypertension compared with DPP4i administration. The results of various sensitivity analyses were consistent with this finding. To our knowledge, this is the first study to demonstrate the possible advantage of SGLT2i in incident hypertension using a large-scale epidemiological database.

Previous RCTs have reported the BP-lowering effects of SGLT2i in individuals with diabetes. The EMPA-REG BP trial showed that the difference of the change in 24-h SBP at 12 weeks from baseline between 25 mg of empagliflozin and placebo was −4.16 mmHg (95% CI: −5.50 to −2.83) among individuals with type 2 diabetes and hypertension [10]. A recent meta-analysis of RCTs observed a reduction in 24-h SBP from baseline of −3.62 mmHg (95% CI: −4.29 to −2.94) in the SGLT-2i group compared to the placebo group [11]. Additionally, the CREDENCE trial showed canagliflozin also reduced the need for initiation of additional antihypertensive medications during the trial in individuals with type 2 diabetes and chronic kidney disease receiving a maximum tolerated or labeled dose of renin angiotensin system blockade (HR, 0.68; 95% CI: 0.61–0.75) [12]. Our study is consistent with these preceding studies and demonstrates the benefits of SGLT2 inhibition.

Our study is distinguishable from preceding studies in the following points, and we believe that this investigation has clinical implications. We studied approximately 20,000 individuals with diabetes using a nationwide real-world dataset and compared approximately 6000 well-balanced pairs of new users of SGLT2i or DPP4i with propensity score matching. Considering the abundant evidence for the BP-lowering effects of SGLT2i, the findings of this study are not surprising. However, it remained unclear whether the moderate BP-lowering (−3 to −4 mmHg) observed in previous studies can reduce even the risk of developing hypertension [10, 11]. We believe that our investigation, which has demonstrated the potential of SGLT2i to reduce incident hypertension, holds clinical relevance. Polypharmacy is common among individuals with diabetes in clinical settings, and our study suggests that the treatment with SGLT2i may negate the need for other antihypertensive medications for individuals with diabetes by preventing the development of hypertension. Indeed, SGLT2i has a range of beneficial effects on the cardiorenal system, lipid metabolism, and uric acid in addition to improved glycemic control [2]. The multifaceted effects of SGLT2i may offer potential benefits for a diverse range of comorbidities present in people with diabetes and could potentially lead to a reduction in the number of prescribed medications. This could serve as strengths of SGLT2i from both public health and economic perspectives.

The mechanisms for reducing BP by the administration of SGLT2i are thought to be multifactorial and have not yet been fully elucidated. Several possible explanations include ameliorated glycemic control, body weight loss, natriuresis, and osmotic diuresis [1, 2]. The cardiovascular and kidney protective actions of SGLT2i have been demonstrated in individuals with and without diabetes [21, 22]. Experimental investigations are required to determine how SGLT2i reduces BP and whether the BP-lowering effects of SGLT2i would differ according to the presence of diabetes. It would also be of value to determine if the lowered risk of developing hypertension remains with longer duration of SGLT2i use.

Our study has inherent limitations, mainly due to the use of our database, as previously described [18, 23]. Because of the observational and retrospective nature of the present study, and despite robust statistical procedures, including propensity score matching and a multitude of sensitivity analyses, the possibility of unmeasured residual confounding effects could not be eliminated. Due to the characteristics of the JMDC Claims Database, it should be noted that the population analyzed in this study had a skewed profile, primarily comprising middle-aged overweight men. Considering this, further investigation using different databases is required to assess the applicability of our findings to other populations, particularly those requiring caution when using SGLT2i (e.g., older individuals, individuals with sarcopenia, individuals who are underweight). Given the recommended BP goals for people having diabetes, the baseline BP values were relatively high. In this study, hypertension was defined based on ICD-10 codes extracted from the JMDC Claims Database. While diagnoses recorded in administrative databases, including the JMDC Claims Database, are often regarded as less validated, a previous investigation has shown a high level of accuracy in the recorded diagnoses in administrative databases in Japan [24–26]. For example, a validation study of the diagnostic codes within the JMDC Claims Database reported a sensitivity and specificity of 74.5% (95% CI: 74.2–74.8%) and 98.2% (95% CI: 98.2–98.3%) for hypertension, respectively [26]. Further, the incident hypertension is determined by either the assignment of a medical diagnosis or the prescription of antihypertensive medications. We should note that this decision is made at the discretion of individual physicians and lacks a standardized criterion. In this study, data on body weight and glycemic control at the end of follow-up, which could have influenced the risk of developing hypertension, were unavailable. Due to the influence of the time period for collecting study participants in this study, many cases were included where some form of glucose-lowering medications were prescribed before the initiation of SGLT2i or DPP4i. In current clinical practice in Japan, SGLT2i or DPP4i are often used as the first-line therapy for the treatment of diabetes. Although our sensitivity analysis excluding individuals who had any antidiabetic medications at the index date showed a consistent result, further investigation is needed into the incidence of hypertension when SGLT2i or DPP4i are used as the first-line therapy. Finally, the dosages of SGLT2i and DPP4i were not considered in this study but are likely relevant to their risk reduction capacity.

### Perspective of Asia

SGLT2i is recognized as important medications in the management of lifestyle-related diseases and subsequent cardiovascular events. Although considering the ethnic differences between Westerners and Asians, this study, which demonstrates the potential of SGLT2i to prevent the risk of hypertension in Japanese individuals with diabetes, is believed to provide very important insights.

## Conclusions

In real-world clinical practice, individuals with diabetes who were newly prescribed SGLT2i showed a reduced risk of developing hypertension compared to those who were newly prescribed DPP4i. The findings of this study shed light on new benefits of SGLT2i as a hypertensive preventive medication.

### Supplementary information

Supplementary Figure 1

Supplementary Table 1
