# Supplementary figures and images for: Comparison of incident hypertension between SGLT2 inhibitors vs. DPP4 inhibitors
Source: Hypertens Res. 2024 Apr 10;47(7):1789–96. doi: 10.1038/s41440-024-01649-z (PMC11224012; doi:10.1038/s41440-024-01649-z)

Supplementary Figure 1. Study design

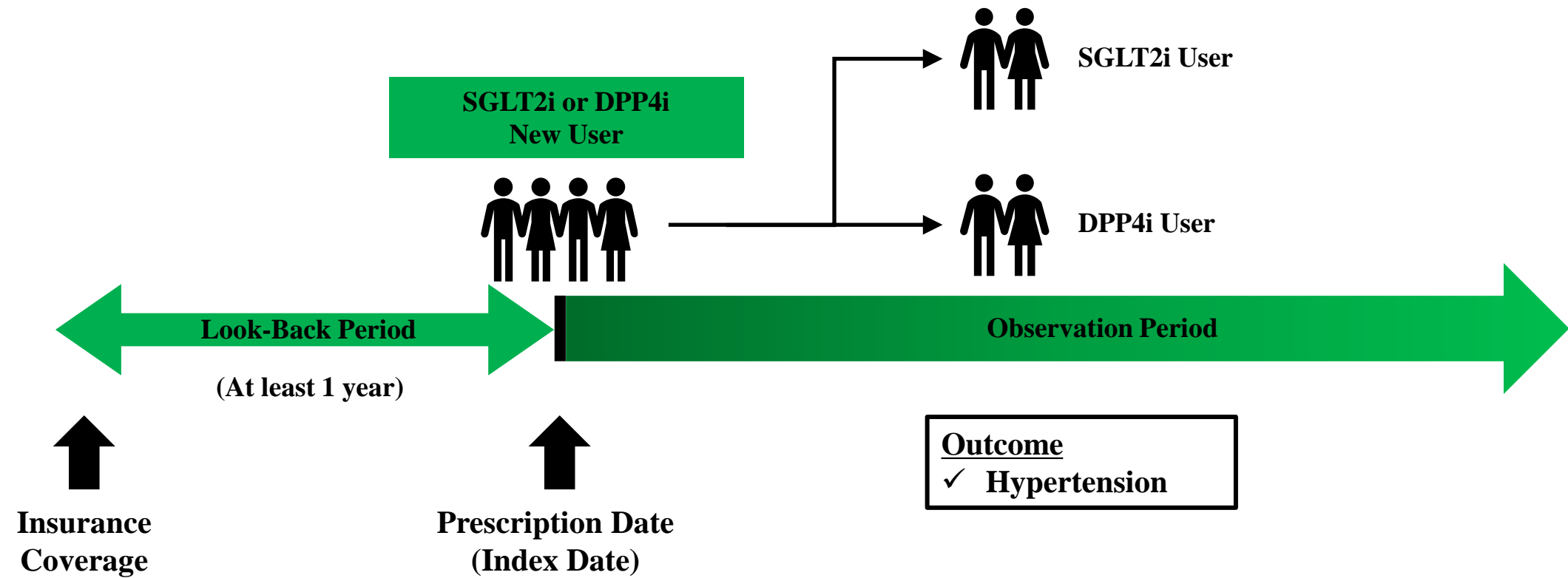

Supplement: Supplementary file 1 — Supplementary Figure 1 [file 41440_2024_1649_MOESM1_ESM.pdf]
