# Supplementary material for: Comparison of incident hypertension between SGLT2 inhibitors vs. DPP4 inhibitors
Source: Hypertens Res. 2024 Apr 10;47(7):1789–96. doi: 10.1038/s41440-024-01649-z (PMC11224012; doi:10.1038/s41440-024-01649-z)
Supplement: Supplementary file 2 — Supplementary Table 1 [file 41440_2024_1649_MOESM2_ESM.docx]

| **Supplementary Table 1. Hazard ratio of developing hypertension among individuals who continued to use SGLT2i or DPP4i for > 3 months** | | | | |
| --- | --- | --- | --- | --- |
|  |  |  |  |  |
|  | Analytical Method | Number | Event | HR (95% CI) |
| DPP4i User | PS Matching | 4,318 | 1,183 | 1 [Reference] |
| SGLT2i User |  | 4,318 | 1,067 | 0.89 (0.82-0.96) |
| We performed Cox proportional hazards regression model to estimate the hazard ratio (HR) and 95% confidence interval (95% CI) of hypertension incidence with sodium-glucose cotransporter 2 inhibitors (SGLT2i) versus dipeptidyl peptidase-4 inhibitors (DPP4i). PS=propensity score. | | | | |

| **Supplementary Table 2. Hazard ratio of developing hypertension among individuals with a prior diagnosis of type 2 diabetes** | | | | |
| --- | --- | --- | --- | --- |
|  |  |  |  |  |
|  | Analytical Method | Number | Event | HR (95% CI) |
| DPP4i User | PS Matching | 3,992 | 1,122 | 1 [Reference] |
| SGLT2i User |  | 3,992 | 1,045 | 0.93 (0.86-1.01) |
| We performed Cox proportional hazards regression model to estimate the hazard ratio (HR) and 95% confidence interval (95% CI) of hypertension incidence with sodium-glucose cotransporter 2 inhibitors (SGLT2i) versus dipeptidyl peptidase-4 inhibitors (DPP4i). PS=propensity score. | | | | |

| **Supplementary Table 3. Hazard ratio of developing hypertension after excluding individuals who had systolic blood pressure ≥ 140 mmHg or diastolic blood pressure ≥ 90 mmHg at the index date** | | | | |
| --- | --- | --- | --- | --- |
|  |  |  |  |  |
|  | Analytical Method | Number | Event | HR (95% CI) |
| DPP4i User | PS Matching | 4,067 | 690 | 1 [Reference] |
| SGLT2i User |  | 4,067 | 618 | 0.88 (0.79-0.99) |
| We performed Cox proportional hazards regression model to estimate the hazard ratio (HR) and 95% confidence interval (95% CI) of hypertension incidence with sodium-glucose cotransporter 2 inhibitors (SGLT2i) versus dipeptidyl peptidase-4 inhibitors (DPP4i). PS=propensity score. | | | | |

| **Supplementary Table 4. Hazard ratio of developing hypertension after redefining the outcome** | | | | |
| --- | --- | --- | --- | --- |
|  |  |  |  |  |
|  | Analytical Method | Number | Event | HR (95% CI) |
| DPP4i User | PS Matching | 5,708 | 992 | 1 [Reference] |
| SGLT2i User |  | 5,708 | 890 | 0.88 (0.80-0.96) |
| We performed Cox proportional hazards regression model to estimate the hazard ratio (HR) and 95% confidence interval (95% CI) of hypertension incidence with sodium-glucose cotransporter 2 inhibitors (SGLT2i) versus dipeptidyl peptidase-4 inhibitors (DPP4i). PS=propensity score. | | | | |

| **Supplementary Table 5. Hazard ratio of developing hypertension after excluding individuals with glucagon-like peptide-1 receptor agonist at the index date** | | | | |
| --- | --- | --- | --- | --- |
|  |  |  |  |  |
|  | Analytical Method | Number | Event | HR (95% CI) |
| DPP4i User | PS Matching | 5,621 | 1,458 | 1 [Reference] |
| SGLT2i User |  | 5,621 | 1,360 | 0.93 (0.86-0.99977) |
| We performed Cox proportional hazards regression model to estimate the hazard ratio (HR) and 95% confidence interval (95% CI) of hypertension incidence with sodium-glucose cotransporter 2 inhibitors (SGLT2i) versus dipeptidyl peptidase-4 inhibitors (DPP4i). PS=propensity score. | | | | |

| **Supplementary Table 6. Hazard ratio of developing hypertension after excluding individuals who had any antidiabetic medications at the index date** | | | | |
| --- | --- | --- | --- | --- |
|  |  |  |  |  |
|  | Analytical Method | Number | Event | HR (95% CI) |
| DPP4i User | PS Matching | 4,037 | 1,201 | 1 [Reference] |
| SGLT2i User |  | 4,037 | 1,123 | 0.93 (0.86-1.01) |
| We performed Cox proportional hazards regression model to estimate the hazard ratio (HR) and 95% confidence interval (95% CI) of hypertension incidence with sodium-glucose cotransporter 2 inhibitors (SGLT2i) versus dipeptidyl peptidase-4 inhibitors (DPP4i). PS=propensity score. | | | | |

| **Supplementary Table 7. Hazard ratio of developing hypertension after overlap weighting** | | | | |
| --- | --- | --- | --- | --- |
|  |  |  |  |  |
|  | Analytical Method | Number | Event | HR (95% CI) |
| DPP4i User | Overlap Weight | 12,419 | 3,439 | 1 [Reference] |
| SGLT2i User |  | 6,181 | 1,479 | 0.93 (0.87-0.99) |
| We performed Cox proportional hazards regression model to estimate the hazard ratio (HR) and 95% confidence interval (95% CI) of hypertension incidence with sodium-glucose cotransporter 2 inhibitors (SGLT2i) versus dipeptidyl peptidase-4 inhibitors (DPP4i). | | | | |
